# Increases in Smoking Cessation Interventions After a Feedback and Improvement Initiative Using Electronic Health Records — 19 Community Health Centers, New York City, October 2010–March 2012

**Published:** 2014-10-17

**Authors:** Sheryl L. Silfen, Shannon M. Farley, Sarah C. Shih, Damon C. Duquaine, Jenna Mandel Ricci, Susan M. Kansagra, Sarah Matthes Edwards, Stephen Babb, Tim McAfee

**Affiliations:** 1 New York City Department of Health and Mental Hygiene; 2 Office on Smoking and Health, National Center for Chronic Disease Prevention and Health Promotion, CDC

Quitting smoking substantially reduces smokers’ risk for smoking-related morbidity and mortality and can increase life expectancy by up to a decade (1). Most smokers want to quit and make at least one medical provider visit annually (2)*.* Health care providers can play an important role in helping smokers quit by documenting patients’ tobacco use, advising smokers to quit, and providing evidence-based cessation treatments or referrals for treatment, but many providers and practices do not regularly take these actions (2). Systems to increase provider screening and delivery of cessation interventions are available (2); in particular, electronic health records (EHRs) can be powerful tools to facilitate increased cessation interventions (3–6). This analysis reports on an EHR-based pay-for-improvement initiative in 19 community health centers (CHCs) in New York City (NYC) that sought to increase smoking status documentation and cessation interventions. At the end of the initiative, the mean proportion of patients who were documented as smokers in CHCs had increased from 24% to 27%, whereas the mean proportion of documented smokers who received a cessation intervention had increased from 23% to 54%. Public health programs and health systems should consider implementing strategies to equip and train clinical providers to use information technology to increase delivery of cessation interventions.

The NYC Department of Health and Mental Hygiene (DOHMH) established the Primary Care Information Project in 2005 to support EHR adoption among primary care practices that provide health care to underserved populations. The Health eQuits program, which was funded by a CDC Communities Putting Prevention to Work grant, targeted CHCs that had implemented EHRs and that were already participating in the Primary Care Information Project with the goal of increasing smoking cessation interventions through incentive payments (7). The program was conducted during October 2010–March 2012, with baseline data collected during October 2009–September 2010. Centers were located in traditionally underserved neighborhoods with a high proportion of Medicaid enrollees, who have a higher smoking prevalence than the general population (18.9% of NYC adults with Medicaid insurance smoke compared with 14.8% of NYC adults overall).* CHCs were required to document smoking status in the EHR at least annually for all patients aged ≥18 years. The initiative included a $20 incentive payment to CHCs (not individual health care providers) for each additional cessation intervention above baseline (capped at $50,000 total).

Qualifying interventions for incentive payments included: physician counseling, prescriptions for cessation medications, or electronic or fax referrals to the New York State quitline. Participating CHCs received quarterly reports based on their EHR data accompanying their payments. For some sites, provider-level reports also were provided upon request. The Health eQuits program manager called or visited practices quarterly to review reports and answer questions. Additional training and support were offered to all CHCs quarterly (7). To assess the initiative’s impact, DOHMH collected data on the unique number of 1) patients, 2) documented smokers, and 3) smokers who received at least one cessation intervention during the 12 months before the start of the program (to create a baseline) and during the 18 months of the program.

The number of unique patients seen by the individual CHCs during the baseline period ranged from 632 to 124,582 (Table). The proportion of Medicaid patients with an office visit at CHCs ranged from 0% to 83%, with a mean of 48% and a median of 49%.

At baseline, the mean documented smoking rate was 24%, with a range of 0% to 75% and a median of 14%; seven of the 19 CHCs reported baseline smoking rates of <10%. In order to be searchable and available for generating reports, information on patients’ smoking status in an EHR was required to be recorded in structured fields. Lower baseline rates of smoking might reflect the failure of CHCs to systematically screen all patients for smoking or the fact that information was not recorded in a reportable format. At the end of the initiative, the mean documented smoking rate was 27%, with a range of 3% to 79% and a median of 17%. Thirteen CHCs showed increases in the proportion of documented smokers, and five CHCs reported smoking rates of <10%.

At baseline, a mean of 23% of documented smokers had received at least one cessation intervention (counseling, cessation medications, and/or referral to the New York State quitline), with a range of 0% to 54% and a median of 16% among the CHCs. At the end of the program, a mean of 54% of documented smokers had received at least one cessation intervention, with a range of 12% to 91% and a median of 58%. Eighteen CHCs showed increases in the proportion of documented smokers who received at least one intervention. As rates of documentation of smoking status improved, intervention rates also increased (Figure). During the 18-month initiative, 36,572 smokers received at least one intervention, compared with only 6,515 smokers during the 12-month baseline period. Over the course of the initiative, NYC paid a total of approximately $220,000 in incentives to the 19 CHCs.

## Discussion

EHRs can facilitate clinical smoking cessation interventions in three ways. First, they can be used to prompt health care providers to screen for and document tobacco use and to intervene with tobacco users by integrating these steps into the clinical workflow (4–6). EHRs also can be used to facilitate provider referral of patients to state quitlines (5,6,8,9), which have broad reach, are effective with diverse populations, and increase quit rates (2).

Second, EHR-generated patient lists (using an EHR registry-like function) can be used to supply providers and practices with rapid feedback on tobacco screening and intervention performance; such feedback can motivate improvement in these areas, especially if performance is compared with other practices and tied to financial or other incentives (3,6,7). Information on performance also can be used to track progress, identify areas where improvement is needed, and ensure that providers and practices receive full credit and reimbursement for their cessation interventions (7).

Third, EHRs can be used to track the impact of clinical cessation and health systems change initiatives on longer-term outcomes in patient populations, including quit rates, smoking rates, and outpatient visits and hospitalizations for smoking-related diseases (3). Such findings can demonstrate to providers, health care systems, and health care policymakers that cessation interventions can reduce smoking prevalence, as well as smoking-related morbidity and health care costs (3). Thus, EHRs have the potential to increase provider screenings and interventions for tobacco use while also making it easier to assess the resulting outcomes (3).

Cigarette smoking remains the leading preventable cause of death and disease in the United States (1). *Healthy People 2020* objectives TU-9 and TU-10 call for increasing tobacco screening and tobacco cessation counseling in health care settings.† The potential role that EHRs can play in increasing cessation interventions likely will grow over time as more physicians and hospitals shift from paper records to EHRs, partly in response to the Centers for Medicare and Medicaid Services Meaningful Use initiative, and also as more smokers gain access to evidence-based cessation treatments under the Affordable Care Act, which requires nongrandfathered private health plans to cover such treatments,§ bars state Medicaid programs from excluding FDA-approved cessation medications from coverage, and requires these programs to provide pregnant women with a comprehensive cessation benefit.¶ Meaningful Use standards require smoking status documentation as a core element to receive Centers for Medicare and Medicaid Services financial incentives.** A recent report found that 72% of U.S. office-based physicians had adopted EHRs as of 2012.††

What is already known on this topic?

Most smokers want to quit and make at least one medical visit each year. Documentation of smoking status and interventions with smokers in health care settings increase quit rates, but many providers and practices do not routinely take these actions.

What is added by this report?

An electronic health record-based pay-for-improvement initiative conducted in 19 community health centers in New York City during October 2010–March 2012 sought to increase smoking status documentation and cessation interventions. At the end of the initiative, the mean proportion of patients who were documented as smokers had increased from 24% to 27%, while the mean proportion of documented smokers who received a cessation intervention increased from 23% to 54%.

What are the implications for public health practice?

Electronic health records have the potential to make it easier for providers to screen for and document tobacco use and to intervene with patients who use tobacco products. In addition, patient lists generated by the electronic health record can be used to offer timely feedback to providers that can motivate better performance, and can also be used to identify sites or issues where improvement is needed. Policymakers might consider harnessing EHRs to support future clinical and health systems cessation initiatives.

The mere adoption of EHRs, however, will not be sufficient to increase the frequency and quality of smoking cessation interventions. Consideration of clinical workflows, incentives, and use of quality improvement approaches are also necessary. In addition, clinical cessation interventions are most effective when they are implemented in conjunction with population-based tobacco control interventions that motivate smokers to quit and support their efforts to do so (1,10). Over the past decade, NYC has implemented several interventions of the latter kind, including smoke-free policies in workplaces and public places, cigarette excise tax increases, and graphic tobacco education mass media campaigns (10). The clinical initiative described in this report complements these efforts by incentivizing CHCs to provide evidence-based cessation assistance to underserved populations.

CHCs serve a high proportion of Medicaid patients, a population known to have a high smoking prevalence. However, over a third of participating clinics initially reported smoking rates of less than 10%, confirming the need for better smoking status screening and documentation to maximize the opportunity for EHRs to have a significant impact on disparities. Increases in observed smoking rates likely are a reflection of increased documentation. Baseline smoking rates reported by CHCs varied widely, which might indicate that some practices, including those with greater proportions of underserved populations and large practices, could require targeted training interventions and other approaches to improve their performance in this area. A separate publication describes the changes implemented in the practice that experienced the greatest increase in smoking cessation interventions.§§

The findings in this report are subject to at least four limitations. First, data were not available for all CHCs on the number of patients who were screened for smoking, the specific cessation interventions delivered, or whether smokers quit. Incentive payments were made for increases above baseline in physician counseling, prescriptions for cessation medications, and electronic or fax referrals to the New York State quitline. Neither the nature nor the effectiveness of the counseling delivered was assessed, and whether prescriptions were filled or quitline referrals led to receipt of quitline services is not known. As a result, the effectiveness of this initiative in reducing smoking cannot be assessed. Future evaluations of similar initiatives should seek to measure these outcomes. However, the types of cessation interventions for which incentive payments were provided have been shown to increase quit rates (2). Second, the intervention was conducted in a single city, so the findings might not be generalizable elsewhere. However, the intervention addressed a diverse, underserved population, and similar results have been reported in other settings (3–6). Third, the effect of implementing EHRs in CHCs was assessed in combination with a financial incentive; therefore, it is uncertain whether the implementation of EHRs alone (without such an incentive) would have yielded similar results. Finally, NYC’s population-based tobacco control interventions could have contributed to the observed increase in clinical cessation interventions by encouraging smokers to ask their health care providers for help quitting.

This analysis suggests that an initiative employing EHRs, feedback to sites, and a monetary incentive can increase clinical cessation interventions with smokers. When totaled across centers, the proportion of all patients with documented smoking status receiving an intervention increased from 20% during the 12-month period preceding the initiative to 62% during the 18-month initiative. The analysis also indicates that data from EHRs can be used to document improvements of this kind, and suggests that EHR data also could be used to capture longer-term outcomes, including quit attempts and quit rates, smoking prevalence, and possibly (with more advanced health information exchanges) smoking disease-related inpatient visits and hospitalizations (3). Return on investment was not calculated for this initiative; an economic evaluation of this sort would be useful.

This initiative could be replicated in other locations, with tailoring to local circumstances as necessary. In addition to facilitating the integration of clinical cessation interventions into routine clinical care, EHRs offer a promising avenue for expanded surveillance and evaluation of the effects of these interventions.

## Figures and Tables

**FIGURE:**
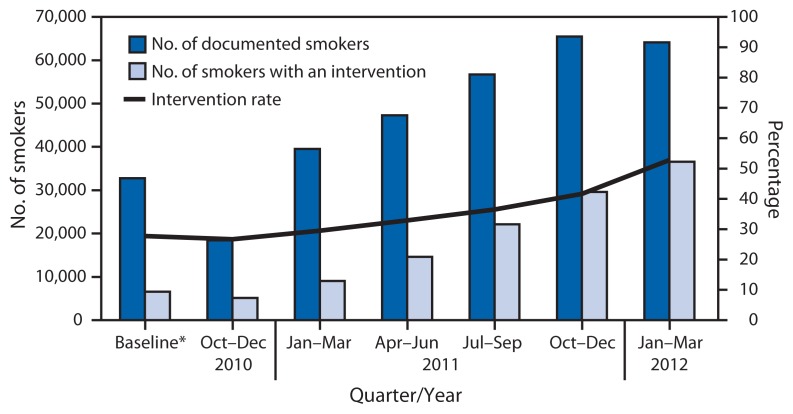
Number of documented smokers, number of smokers with an intervention, and intervention rate, by quarter — 19 community health centers, New York City, October 2010–March 2012 * Baseline data were collected during October 2009–September 2010.

**TABLE:** Smoking documentation and intervention before and after a pay-for-improvement initiative using electronic health records (EHRs) — 19 community health centers, New York City, October 2010–March 2012

Reported practice characteristics at baseline*					Unique patients		Documented smokers				Smokers with at least one intervention				
	
							Baseline*		End†		Baseline*		End†		Percentage-point change from baseline to end
					
Practice ID no.	No. of mos. using EHR	No. of sites	No. FTE providers	Medicaid (%)	Baseline* No.	End† No.	No.	(%)	No.	(%)	No.	(%)	No.	(%)	
1	22	9	86	(2)	45,998	26,732	5,889	(13)	3,351	(13)	805	(14)	412	(12)	(−2)
2	24	3	23	(41)	43,468	47,268	32	(<1)	7,744	(16)	0	(0)	1,240	(16)	(16)
3	11	4	11	(64)	4,748	5,672	928	(20)	1,120	(20)	204	(22)	292	(26)	(4)
4	32	13	45	(57)	27,420	38,680	3,708	(14)	1,304	(3)	488	(13)	380	(29)	(16)
5	22	8	48	(67)	26,328	31,072	444	(2)	2,248	(7)	48	(11)	680	(30)	(19)
6	10	1	14	(57)	5,680	7,448	488	(9)	1,344	(18)	76	(16)	424	(32)	(16)
7	18	2	21	(80)	12,412	13,844	0	(0)	672	(5)	0	(0)	304	(45)	(45)
8	35	5	6	(68)	1,592	2,264	1,008	(63)	1,180	(52)	248	(25)	580	(49)	(24)
9	24	6	4	(48)	5,340	5,484	1,820	(34)	2,072	(38)	928	(51)	1,204	(58)	(7)
10	28	1	5	(49)	2,324	2,108	336	(14)	640	(30)	84	(25)	372	(58)	(33)
11	17	1	12	(83)	3,056	3,160	2,292	(75)	2,508	(79)	108	(5)	1,552	(62)	(57)
12	17	1	6	(63)	868	916	436	(50)	496	(54)	68	(16)	308	(62)	(46)
13	27	1	2	(20)	632	932	112	(18)	104	(11)	60	(54)	68	(65)	(11)
14	11	24	33	(46)	29,292	11,572	140	(<1)	1,079	(9)	8	(6)	744	(69)	(63)
15	24	22	464	(43)	124,582	202,450	10,129	(8)	25,536	(13)	854	(8)	21,620	(85)	(76)
16	95	4	61	(42)	NA	NA	1,384	(NA)	2,692	(NA)	652	(47)	1,924	(71)	(24)
17	8	4	8	(0)	2,088	1,800	1,068	(51)	988	(55)	448	(42)	708	(72)	(30)
18	31	5	16	(75)	3,912	3,960	2,220	(57)	2,372	(60)	1,176	(53)	1,980	(83)	(30)
19	81	4	NA	(0)	39,276	39,545	969	(2)	1,955	(5)	260	(27)	1,780	(91)	(64)
*Mean*	*28*	*6*	*48*	*(48)*	*21,056*	*24,717*	*1,758*	*(24)*	*3,127*	*(27)*	*343*	*(23)*	*1,925*	*(54)*	*(31)*
*Median*	*24*	*4*	*15*	*(49)*	*5,510*	*6,560*	*969*	*(14)*	*1,344*	*(17)*	*204*	*(16)*	*680*	*(58)*	*(42)*
**Total**	**NA**	**118**	**865**	**NA**	**379,016**	**444,907**	**33,403**	**(9)**	**59,405**	**(13)**	**6,515**	**(20)**	**36,572**	**(62)**	**(42)**

**Abbreviations:** NA = not available (means, medians, and totals do not include these missing data); FTE = full-time equivalent.

* Baseline data were collected during October 2009–September 2010.

† Centers provided data for the 18-month duration of the program.

## Footnotes

* Additional information available at https://a816-healthpsi.nyc.gov/epiquery.

† Additional information available at http://www.healthypeople.gov/2020/topics-objectives/topic/tobacco-use/objectives.

§ Additional information available at http://www.dol.gov/ebsa/faqs/faq-aca19.html.

¶ Additional information available at http://docs.house.gov/energycommerce/ppacacon.pdf.

** Additional information available at http://www.cms.gov/regulations-and-guidance/legislation/ehrincentiveprograms/meaningful_use.html.

†† Additional information available at http://www.cdc.gov/nchs/data/databriefs/db111.htm.

§§ Additional information available at http://millionhearts.hhs.gov/docs/ss_ny.pdf.

## References

[1] US Department of Health and Human Services (2014). The health consequences of smoking—50 years of progress: a report of the Surgeon General.

[2] Fiore MC, Jaen CR, Baker TB (2008). Treating tobacco use and dependence: 2008 update Clinical practice guideline.

[3] Land TG, Rigotti NA, Levy DE, Schilling T, Warner D, Li W (2012). The effect of systematic clinical interventions with cigarette smokers on quit status and the rates of smoking-related primary care office visits. PLoS One.

[4] Boyle R, Solberg L, Fiore M (2011). Use of electronic health records to support smoking cessation. Cochrane Database Syst Rev.

[5] Greenwood DA, Parise CA, MacAller TA (2012). Utilizing clinical support staff and electronic health records to increase tobacco use documentation and referrals to a state quitline. J Vasc Nurs.

[6] Bentz CJ, Bayley KB, Bonin KE (2007). Provider feedback to improve 5A’s tobacco cessation in primary care: a cluster randomized clinical trial. Nicotine Tob Res.

[7] Duquaine D, Farley SM, Sacks R, Mandel-Ricci J, Silfen SL, Shih SC (2014). Designing a quality improvement program with electronic health records: New York City’s Health eQuits. Am J Med Qual.

[8] Warner DD, Land TG, Rodgers AB, Keithly L (2012). Integrating tobacco cessation quitlines into health care: Massachusetts, 2002–2011. Prev Chronic Dis.

[9] Vidrine JI, Shete S, Cao Y (2013). Ask-Advise-Connect: a new approach to smoking treatment delivery in health care settings. JAMA Intern Med.

[10] Frieden TR, Mostashari F, Kerker BD, Miller N, Hajat A, Frankel M (2005). Adult tobacco use levels after intensive tobacco control measures: New York City, 2002–2003. Am J Public Health.

